# *QuickStats:* Percentage* of Adults Aged ≥45 Years Who Use a Hearing Aid,^†^ by Sex and Age Group — National Health Interview Survey, United States, 2021^§^

**DOI:** 10.15585/mmwr.mm7206a5

**Published:** 2023-02-10

**Figure:**
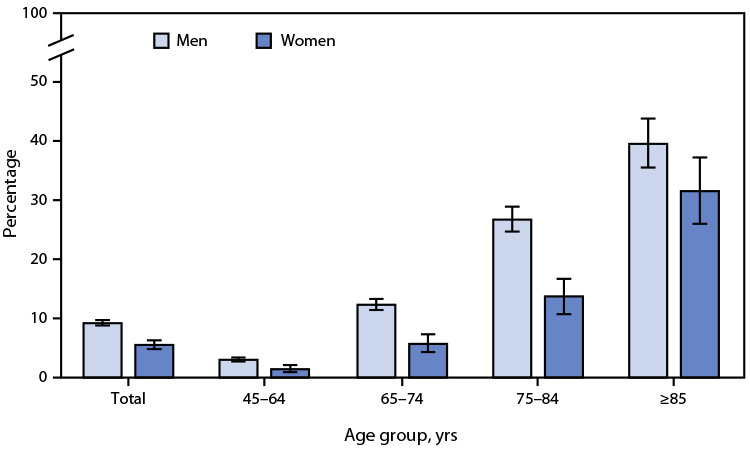
In 2021, among adults aged ≥45 years, men were more likely to use a hearing aid than were women (9.2% versus 5.5%). This pattern was found in all age groups: 3.0% of men versus 1.4% of women among those aged 45–64 years, 12.3% versus 5.7% among those aged 65–74 years, 26.7% versus 13.7% among those aged 75–84 years, and 39.5% versus 31.5% among those aged ≥85 years. Among both men and women, the percentage who use a hearing aid increased with age.

**Sources:** National Center for Health Statistics, National Health Interview Survey, 2021 (https://www.cdc.gov/nchs/nhis.htm); NCHS data brief, no. 414, 2021. https://www.cdc.gov/nchs/products/databriefs/db414.htm

